# A Practical Treatise on Diseases of the Skin

**Published:** 1889-04

**Authors:** 


A PRACTICAL TREATISE ON DISEASES OF THE SKIN, FOR THE USE OF STUDENTS AND PRACTITIONERS. Second edition, revised and enlarged, by JAMES NEVINS HYDE, A. M., M. D. Philadelphia, Lea Bros. & Co., 1888, pp. xix, 676.

A very excellent work, and one to be recommended to both "students and practitioners." In these days of compends and aids without number, it is refreshing to meet with a book like this on a special subject, which is not pedantic and prosily historical on the one hand, or so short, sketchy and incomplete on the other, that the reader and student feels like the child who complained of a noted Georgia preacher: "He preached so long I got tired and sleepy, and so loud I couldn't go to sleep!" Too many books on special subjects are so tiresome that they make one tired, but are so aggravating that they stir up his anger. Hyde, on the skin, is both interesting and instructive. It is written in a clear, limpid style, with no unnecessary padding, and yet with sufficient fullness to make every point perfectly plain. The *treatment* is given in each case very fully, the author leaving no doubt as to what he prefers, yet also accompanying are found the opinions of the best authorities. Great credit is due for the simplification of nomenclature and classification which adds much to the comfort and instruction of the seeker for knowledge. There is no other book on skin diseases but this which combines the qualities of fullness, accuracy, reliability and proper condensation, and presents them in such convenient and attractive form.

